# Nanomedicine in Oncocardiology: Contribution and Perspectives of Preclinical Studies

**DOI:** 10.3389/fcvm.2021.690533

**Published:** 2021-06-30

**Authors:** Gabriel Silva Marques Borges, Eduardo Burgarelli Lages, Pierre Sicard, Lucas Antônio Miranda Ferreira, Sylvain Richard

**Affiliations:** ^1^Departamento de Produtos Farmacêuticos, Faculdade de Farmácia, Universidade Federal de Minas Gerais, Belo Horizonte, Brazil; ^2^PhyMedExp, Université de Montpellier, INSERM, CNRS, Montpellier, France; ^3^IPAM, BioCampus, CNRS, INSERM, Université de Montpellier, Montpellier, France

**Keywords:** anticancer drugs, cardiotoxicity, nanoformulations, non-invasive imaging, echography, photoacoustic, small animals

## Abstract

Cancer and cardiovascular diseases are the leading causes of death and morbidity worldwide. Strikingly, cardiovascular disorders are more common and more severe in cancer patients than in the general population, increasing incidence rates. In this context, it is vital to consider the anticancer efficacy of a treatment and the devastating heart complications it could potentially cause. Oncocardiology has emerged as a promising medical and scientific field addressing these aspects from different angles. Interestingly, nanomedicine appears to have great promise in reducing the cardiotoxicity of anticancer drugs, maintaining or even enhancing their efficacy. Several studies have shown the benefits of nanocarriers, although with some flaws when considering the concept of oncocardiology. Herein, we discuss how preclinical studies should be designed as closely as possible to clinical protocols, considering various parameters intrinsic to the animal models used and the experimental protocols. The sex and age of the animals, the size and location of the tumors, the doses of the nanoformulations administered, and the acute vs. the long-term effects of treatments are essential aspects. We also discuss the perspectives offered by non-invasive imaging techniques to simultaneously assess both the anticancer effects of treatment and its potential impact on the heart. The overall objective is to accelerate the development and validation of nanoformulations through high-quality preclinical studies reproducing the clinical conditions.

## Introduction

Recent advances in the treatment of cancers have improved patient care and cure rates. Cancer, once fatal, is now emerging as a chronic disease, often at the cost of cardiovascular complications. Moreover, cardiovascular diseases (CVD) are more common and more severe in cancer patients (1–5). In this context, the development of novel treatments with antineoplastic agents still raises concerns about undesirable effects at the acute phase of the treatment and potentially during long-term therapy (6). Therefore, the management of cancer patients has moved from general cardiology to a specialized discipline, oncocardiology, with in-depth cardiovascular monitoring at each stage of cancer therapy (1, 7). Among various areas that can help solve these problems, nanotechnology is being increasingly investigated in preclinical studies to improve anticancer treatments and reduce their cardiotoxicity. This review will discuss the potential of nanocarriers to optimize the use of anticancer drugs through tailored preclinical approaches that can help understand/control their adverse effects and how preclinical models can be better designed to represent an oncocardiology approach truly.

## Reducing Cardiotoxicity of Anticancer Drugs Through Nanotechnology

Cardiotoxicity is a broad term that embraces toxic effects on cardiac structure, morphology, rest parameters, and the dynamic function of the heart. Cytotoxic drugs and targeted therapies used to treat cancer, including classic chemotherapeutic agents, tyrosine kinase inhibitors, antiangiogenic drugs, and immunotherapy, often impair the cardiovascular system (8). Of note, cancer itself may promote CVD (6).

The cardiotoxicity evoked by conventional chemotherapy is widely known and occur through multiple mechanisms, including overproduction of free radical species, inhibition of topoisomerase 2β causing DNA double-strand breaks and activation of apoptosis, profound changes in the transcriptome leading to the generation of reactive oxygen species, and inhibition of VEGF receptors causing endothelial dysfunction and vascular injury. In turn, the novel immunotherapeutic agents can lead to the inhibition of the Human Epithelial Receptor type 2 (HER2), impairing the cell signaling in cardiomyocytes; and the inhibition of programmed cell death proteins (PD-1) causing inflammatory infiltrates in the heart tissue (8–10). This cardiotoxicity can lead to bradycardia (paclitaxel and thalidomide), promote QT interval prolongation and arrhythmias (amsacrine and anthracyclines), evoke myocardial ischemia via coronary vasospasm (anti-metabolites and 5-fluorouracil), impair the left ventricle function (anthracyclines, tyrosine kinase inhibitors, alkylating agents, and cisplatin), and evoke myocarditis, pericarditis, and heart failure (immunotherapy) (6, 11, 12).

The objective of cardioprotection is to limit cardiac damage while maintaining antineoplastic efficacy (8). In the clinics, combinations with drugs like statins, antiarrhythmic, beta-blockers, calcium-channel inhibitors, and ACE (angiotensin-converting enzyme) inhibitors are some of the strategies (13). Interestingly, preclinical and clinical studies show that nanotechnology can also lead to cardioprotection in cancer treatment (14).

**Figure 1:**
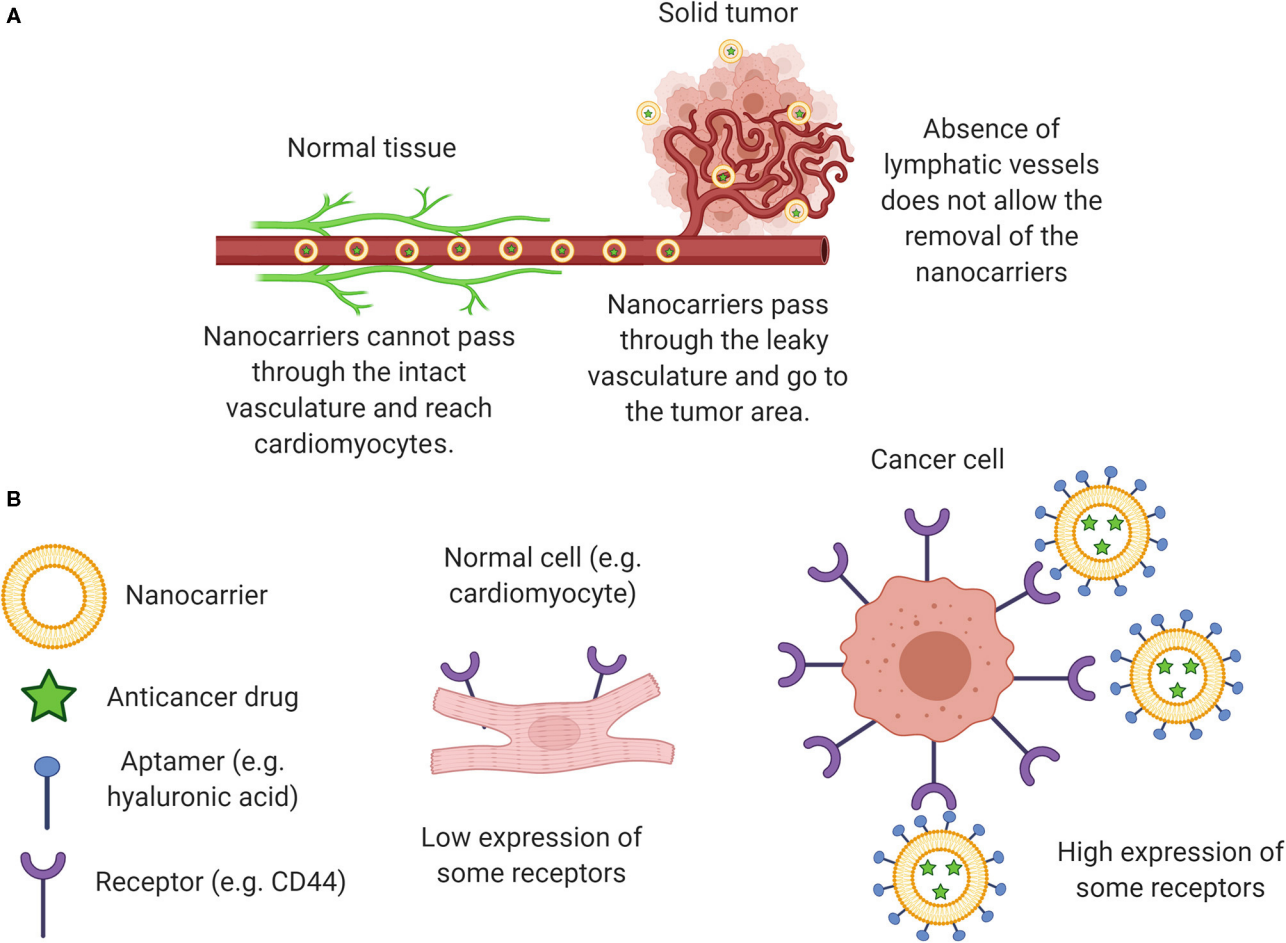
**(A)** Representation of the EPR effect with the nanocarriers accumulating within the solid tumor and not within the cardiomyocytes; **(B)** Nanocarriers with active targeting, favoring the specificity for cancer cells instead of cardiomyocytes. EPR effect, enhanced permeability and retention effect.

The basic concept of nanomedicine is to alter both pharmacokinetics and biodistribution of nanoencapsulated drugs, increasing their accumulation in the tumor site and decreasing their delivery to non-target organs, such as the heart. Passive and active targeting mechanisms can enable nanocarriers to reach the tumor site efficiently. The passive mechanism is based on the Enhanced Permeability and Retention (EPR) effect, which leads to the accumulation of nanostructures in the tumor site due to the characteristic leaky vasculature and absence of lymphatic drainage in the tumor microenvironment (Figure 1A) (15). In turn, active targeting involves the attachment of high-affinity ligands to tumor cells on the surface of the nanocarriers. The idea is to use ligands for which cancer cells express a high number of specific receptors, whereas normal cells express very few. A wide variety of ligands have been used for this purpose, including folic acid, hyaluronic acid, transferrin, among others (Figure 1B) (15, 16). Another interesting approach to evoke cardioprotection through nanotechnology is the co-delivery of antineoplastic drugs and cardioprotective agents (17). For example, the co-encapsulation of DOX with curcumin, quercetin, or docosahexaenoic acid within the same nanocarrier has been proposed in several nanoscale systems (18–20).

In 1995, Doxil® (pegylated liposomal DOX) was the first nanocarrier approved for clinical use (21, 22). This formulation was designed to reduce DOX toxicity while preserving its antitumor efficacy by altering its tissue distribution and pharmacokinetics (23). Several randomized controlled trials have demonstrated a reduction in the risk of clinical cardiotoxicity for liposomal DOX-based chemotherapy compared to free DOX (24, 25). At present, various commercial formulations of nanocarriers have been approved or are under clinical trial for cancer therapy. These nanocarriers deliver classic chemotherapeutics, such as isolated paclitaxel, irinotecan, the synergistic combination of daunorubicin and cytarabine, and recent innovative treatments for T-cell cancer immunotherapy (26, 27).

## Considerations About Preclinical Studies of Nanocarriers

**Figure 2:**
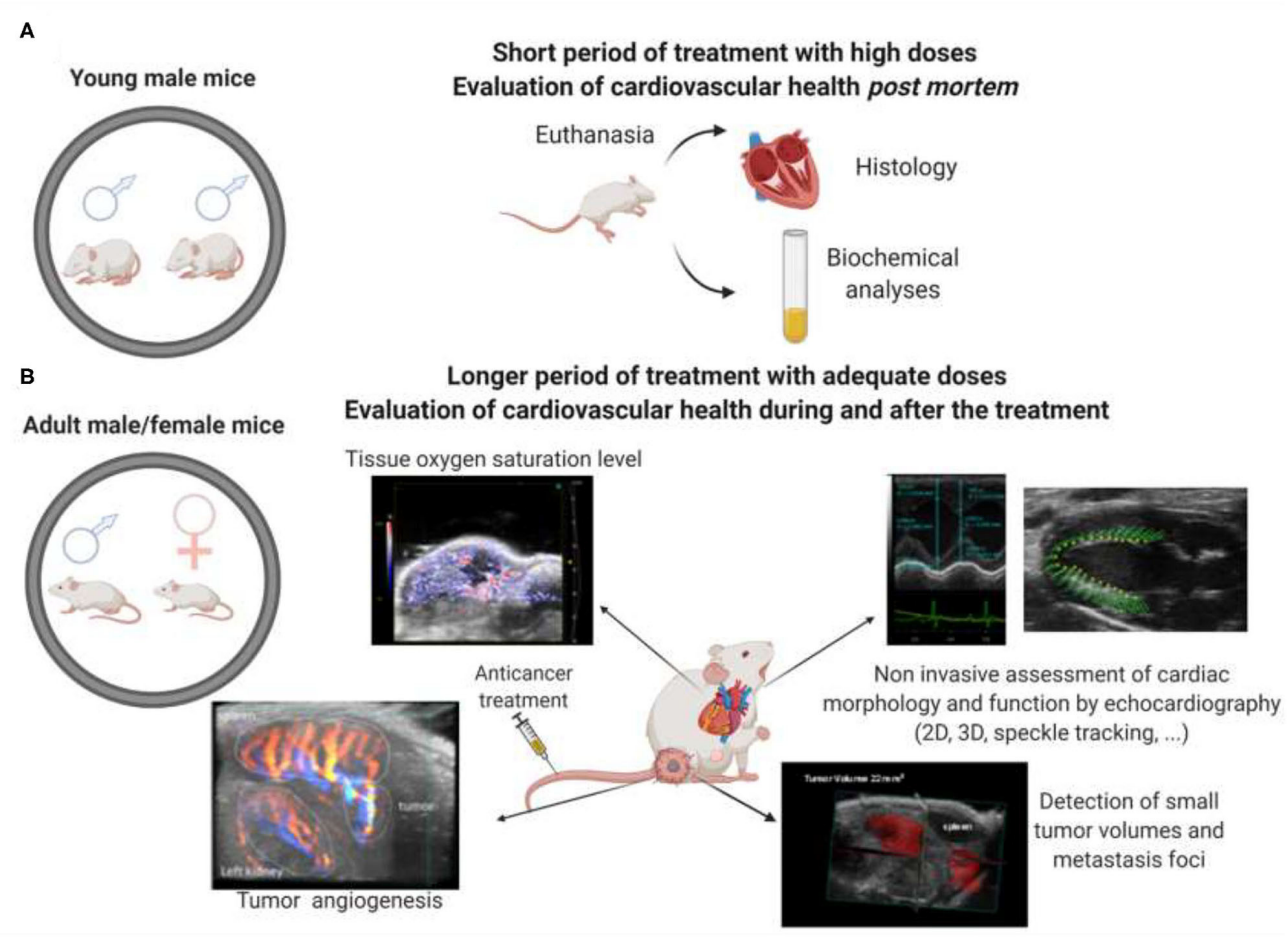
**(A)** Representation of how anticancer animal studies are generally conducted up to now, with cardiotoxicity evaluation being conducted only after euthanasia of the animals, with high doses administered of the nanocarriers and the use of only male and very young animals; **(B)** Suggestion of how to conduct animal studies with nanocarriers using both female and male older mice; evaluation of cardiotoxicity during and after the treatment administered with lower doses of the nanocarriers. The ultrasound-based techniques allow the simultaneous evaluation of antitumor activity and cardiotoxicity.

The evaluation of the cardiotoxicity of nanostructures can be performed both *in vitro* and *in vivo*. Human pluripotent stem cell-derived cardiomyocytes (hiPSC-CM) emerge as a practical approach for the early *in vitro* screening of cardiotoxicity of new drugs (28–30). However, investigations on animal models are still required to develop their use in clinical practice despite some limitations. Nonetheless, some characteristics of these models, such as age, gender, drug doses, and tumor models, are often not correctly considered (Figure 2A). We will discuss these aspects in the following sections.

### Age and Gender

Most cancer patients are more than 60 years old, with aging being a critical predisposition factor for cancer and CVD (5). Despite these predictions, many preclinical cancer studies use mice whose age correlates with that of adolescents (Figure 2A), a period of life when cancer and cardiovascular events are fortunately rare. The physiological differences between young and old animals are significant, and one example is related to the EPR effect. Tumor-bearing young animals may exhibit a more pronounced EPR effect than older animals since older subjects present a lower tumor angiogenesis rate (31). This issue makes less probable the formation of blood vessels with fenestrations allowing the higher permeation of nanocarriers by tumor tissues in young animals compared to older subjects (32). Cardiovascular adverse effects of therapeutics can also be underestimated in young animals since the hearts of these animals have a higher regenerative potential than the hearts of old animals (33, 34). Therefore, the widespread use of very young animals is a serious flaw that impairs the translation of the results from preclinical to clinical studies.

Regarding gender, men are the most affected by almost all types of cancer, with a few exceptions, such as anorectal, gallbladder, thyroid, and breast cancer. However, we expect the number of women affected by neoplasms to increase. Therefore, it is essential to evaluate some risk factors affecting women when they are gender-specific or affect women more intensely, such as menopause, smoking, diabetes, and pregnancy (35). Additionally, drug treatments for CVD may not be the same due to physiological differences between the sexes (36). As an example, anthracyclines-induced cardiotoxicity can be less pronounced in women due to some benefits provided by estrogens (37). Despite all this, most studies use only male animals (Figure 2A), which does not reveal whether a particular cancer treatment works in the same way in women or whether its side effects are the same. Overall, preclinical cancer research should include sex as a biological variable in all investigations (35) (Figure 2B).

### Tumor Models

Regarding the formation of tumors in animal models, there are issues such as their location and size. The orthotopic localization provides the appropriate microenvironment to mimic metastases caused by a specific tumor (38). However, the establishment of orthotopic tumors is an exception, and most studies involve subcutaneous inoculation of tumor cells in the flank of the animals. In addition, the sizes of the tumors implanted subcutaneously are generally substantial to allow their measurements through calipers. Consequently, the relationship between tumor weight/animal weight becomes much higher than that observed in humans, leading to errors when interpreting the results. Human tumors treated with nanomedicine are usually in the order of a few grams, so the tumor/body weight relationship is insignificant. In mice, on the other hand, tumors can reach up to 10% of the animal's weight. Large size is one factor that causes the EPR effect of nanomedicines to be overestimated in animals, as larger tumors can promote higher colloidal buildup (39). Ideally, the tumors should not exceed few millimeters to respect better the relationship between tumor and body weight observed in humans. Additionally, small tumors allow more prolonged treatment and animal monitoring periods, as animals must be euthanized when the tumors reach high volumes.

### Administered Doses of Nanomedicines

It is essential to administer, to the animals, doses related to the plasma blood concentrations found in the clinics (40). However, the choice of dosage in efficacy and toxicity preclinical studies is often neglected, compromising the success of these therapies in humans. First, very high doses are often utilized in animal studies to reduce large volume tumors. Therefore, the use of lower doses relies on the inoculation of smaller volume tumors in the animals. In addition, the maximum tolerated doses in mice are generally higher than in humans (40). Thus, the doses used in animal models can sometimes not be applied to human studies, making the clinical translation of these formulations limited. In the specific case of nanocarriers, their reduced systemic toxicity allows the administration of even higher doses to the animals (41). Nonetheless, one should stress that reduced acute toxicity does not always mean reduced chronic toxicity (42, 43).

### Evaluation of Chronic Cardiotoxic Events

An important concept to apply in preclinical studies with nanocarriers is the follow-up of animals after anticancer treatment. Many significant cardiac side effects often occur later, even years after antineoplastic therapy (44). However, animals are often euthanized rapidly after the treatment is completed (39). Additionally, most preclinical studies using nanotechnology for the treatment of cancer only perform post-mortem cardiovascular assessments. These analyses are based mainly on the biochemical quantification of certain enzymes and the histology of the heart. Although the information obtained by these methods is valid, this does not reflect an oncocardiology approach, as they do not monitor cardiovascular health during cancer treatment, as practiced in human patients. Moreover, studies that focus on cardiovascular toxicity are usually done in healthy animals rather than in animals with tumors, with some drawbacks because the toxic effects may differ in non-tumor subjects and tumor carriers (45).

The following of laboratory animals through more extensive periods, although desirable, can be costly and laborious for the researchers. One exciting option is the use of domestic animals such as dogs and cats. These species spontaneously develop tumors, and the owners maintain their pets through the years after the diagnosis. These animals can participate in experimental therapies, close to clinical trials, evaluating tumor regression and cardiovascular health in partnership with veterinaries (46, 47).

### Simultaneous Evaluation of Antitumor and Cardiotoxic Effects

Anticancer activity and cardiotoxicity should, when possible, be considered simultaneously (Figure 2B). A holistic approach could help in the exploitation of divergent results. For example, the combination of DOX, trastuzumab, and taxanes is very effective in treating breast cancer, but it produces severe synergistic cardiotoxic events in female patients (48, 49). Additionally, premature aging induced by cancer treatment may contribute to chronic health problems in cancer survivors (7). Moreover, the nanocarriers can evoke cardiotoxicity *per se*. One example is the immune reactions to polyethylene glycol molecules attached to the surface of nanocarriers, known as complement activation-related pseudoallergy, which can even lead to a patient's death due to arrhythmias, ventricular fibrillation, and cardiac arrest (50). Moreover, oncolytic viruses, an increasingly used drug delivery vehicle, can replicate themselves in non-targeted tissues, evoking inflammation of the heart, leading to myocarditis (51).

Few preclinical studies have monitored both antitumor activity and cardiac function, in the same animal model, during and after the treatment with nanocarriers. To the best of our knowledge, the three studies presented below are the only ones to have evaluated both simultaneously, all of them using DOX-loaded nanocarriers. DOX loaded in folate-coated liposomes, injected intravenously in breast tumor-bearing mice, showed higher antitumor activity than free DOX and reduced cardiotoxicity (52). Similar findings were found after administering a liposomal formulation loading DOX to colon tumor-bearing mice (53). In addition, polymeric magnetic nanoparticles co-encapsulating DOX and verapamil (a calcium channel blocker) with active targeting of RGD (arginyl glycyl aspartic acid) peptide improved antitumor activity and decreased cardiotoxicity compared to free DOX (54). All these studies evaluated cardiotoxicity during treatment by analysis of the ECG and the QT interval. Imaging techniques have not been used, although they can provide essential data. To monitor smaller and orthotopic tumors and cardiotoxicity more precisely, during and after treatment, the use of non-invasive imaging techniques should be encouraged (Figure 2B).

### Ultrasound Techniques to Assess the Antitumor Activity and Cardiotoxicity of Nanomedicines

Different imaging techniques such as magnetic resonance imaging (MRI), positron emission tomography (PET), SPECT (single photon emission tomography), and computed tomography (X - computer tomography) can simultaneously assess antitumor activity and cardiotoxicity evoked by anticancer treatments (55–57). These imaging techniques allow the early detection and monitoring of a wide range of cardiotoxic events, including systolic and diastolic dysfunction, morphological changes, coronary artery, valve, and pericardial diseases resulting from cancer treatment (56). However, the significant problems with these techniques are their high cost, long scanning time (30–60 min/animal), and radiation exposure (PET, SPECT, and CT) (58).

Real-time image acquisition, ease of data interpretation, and low cost make ultrasound imaging a promising and suitable approach, compared to other techniques, to monitor cardiotoxic events in preclinical studies (59–63). Ultrasound allows rapid longitudinal monitoring (< 10 min/animal for each control) of tumor and cardiac function before, during, and after anticancer treatment. This opportunity is of particular interest for monitoring tumor growth, as it is possible to monitor the animals for more extended periods (64, 65) and to assess the volume of implanted orthotopic tumors (66). This approach also makes it possible, on a practical level, to homogenize the different study groups as a function of a reference tumor volume. This possibility reduces the number of animals used while minimizing the variability in the distribution within each experimental group.

Finally, photoacoustic imaging (PAI) is another promising ultrasound-based technique. It is a relatively new hybrid modality that combines optical imaging contrast with the spatial resolution of ultrasound (67, 68). PAI provides high-resolution images of optical absorption in deep tissues, allowing visualization of angiogenesis, tissue oxygen saturation, and metabolic or inflammatory parameters (69–71). In addition, PAI has shown promise in detecting, diagnosing, and guiding cancer treatment due to its ability to detect or activate specific nanostructures to enhance contrast (72, 73).

## Concluding Remarks

Clinics have widely demonstrated the concept that nanomedicines improve the therapeutic index of anticancer drugs (15, 74). The advances in nanoscale delivery systems offer great hope to increase the efficacy of drugs in a targeted manner and overcome the toxicity limitations associated with conventional free drug delivery. Translating the results from preclinical studies to more clinically relevant models is an urgent demand and a significant challenge to alleviate the burden of the undesirable cardiotoxic effects of anticancer drugs (41, 75). Considering the advantages and limitations of animal models and the peculiarities of nanoformulations, appropriate techniques for monitoring antitumor efficacy and cardiovascular health will be essential to practice the best oncocardiology approach.

## Author Contributions

All authors contributed to the writing and editing of the manuscript.

## Conflict of Interest

The authors declare that the research was conducted in the absence of any commercial or financial relationships that could be construed as a potential conflict of interest.
